# Characteristics and Outcome of SARS-CoV-2 Infection in Cancer Patients

**DOI:** 10.1093/jncics/pkaa090

**Published:** 2021-01-06

**Authors:** Clémence Basse, Sarah Diakite, Vincent Servois, Maxime Frelaut, Aurélien Noret, Audrey Bellesoeur, Pauline Moreau, Marie-Ange Massiani, Anne-Sophie Bouyer, Perrine Vuagnat, Sandra Malak, François-Clément Bidard, Dominique Vanjak, Irène Kriegel, Alexis Burnod, Geoffroy Bilger, Toulsie Ramtohul, Gilles Dhonneur, Carole Bouleuc, Nathalie Cassoux, Institut Curie COVID Group,, Xavier Paoletti, Laurence Bozec, Paul Cottu

**Affiliations:** 1 Department of Medical Oncology, Institut Curie, Paris & Saint Cloud, France; 2 Department of Radiology, Institut Curie, Paris, France; 3 Department of Drug Development and Innovation, Institut Curie, Paris & Saint Cloud, France; 4 Université Versailles Saint-Quentin, Université Paris-Saclay, Saint Cloud, France; 5 Infectious Diseases Unit, Institut Curie, Paris & Saint Cloud, France; 6 Department of Anesthesia, Intensive Care and Pain Medicine, Institut Curie, Paris & Saint Cloud, France; 7 Supportive and Palliative Care Department, Institut Curie, Paris & Saint-Cloud, France; 8 Department of Surgical Oncology, Institut Curie, Paris & Saint-Cloud, France; 9 Université de Paris, Paris, France

## Abstract

**Background:**

Concerns have emerged about the higher risk of fatal coronavirus disease 2019 (COVID-19) in cancer patients. In this article, we review the experience of a comprehensive cancer center.

**Methods:**

A prospective registry was set up at Institut Curie at the beginning of the COVID-19 pandemic. All cancer patients with suspected or proven COVID-19 were entered and actively followed for 28 days.

**Results:**

Among 9842 patients treated at Institut Curie between March 13 and May 1, 2020, 141 (1.4%) were diagnosed with COVID-19, based on reverse transcription polymerase chain reaction testing and/or computerized tomography scan. In line with our case mix, breast cancer (40.4%) was the most common tumor type, followed by hematological and lung malignancies. Patients with active cancer therapy or/and advanced cancer accounted for 87.9% and 68.9% of patients, respectively. At diagnosis, 78.7% of patients had COVID-19–related symptoms, with an extent of lung parenchyma involvement inferior to 50% in 95.8% of patients. Blood count variations and C-reactive protein elevation were the most common laboratory abnormalities. Antibiotics and antiviral agents were administered in 48.2% and 6.4% of patients, respectively. At the time of analysis, 26 patients (18.4%) have died from COVID-19, and 100 (70.9%) were cured. Independent prognostic factors at the time of COVID-19 diagnosis associated with death or intensive care unit admission were extent of COVID-19 pneumonia and decreased O_2_ saturation.

**Conclusions:**

COVID-19 incidence and presentation in cancer patients appear to be very similar to those in the general population. The outcome of COVID-19 is primarily driven by the initial severity of infection rather than patient or cancer characteristics.

Since December 2019, the world has been faced with the emergence of coronavirus disease 2019 (COVID-19), caused by a novel beta-coronavirus called severe acute respiratory syndrome coronavirus 2 (SARS-CoV-2) (1). The first cases of pneumonia were reported in Wuhan City, China, in December 2019 (2) and were rapidly followed by worldwide dissemination. The World Health Organization consequently declared COVID-19 (also called SARS-Cov-2 infection) a public health emergency of international concern (3). COVID-19 has now affected more than 4 million people worldwide and has already been responsible for more than 250 000 deaths (4). France has the 6th highest number of cases of COVID-19 in the world, with almost 170 000 confirmed cases and more than 29 000 deaths (5). The Paris area (Ile-de-France) is the epicenter of the epidemic in France (6).

Cancer patients could represent a high-risk population in this global crisis. Based on a series of 44 672 confirmed cases in China, Wu, and McGoogan reported a 2.3% death rate in the general population vs 5.6% among cancer patients (7). Several studies have simultaneously reported that preexisting comorbidities (including cancer) constitute a risk factor associated with severe COVID-19 infection and intensive care unit (ICU) admission (8,9). COVID-19 appears to be more frequent among cancer patients, with an overrepresentation of cancer patients in the confirmed COVID-19 population compared with the general population in China (10,11). Initial retrospective cohorts of cancer patients with COVID-19 in China were characterized by a high rate of severe presentation (39% to 54% of cases) and a high mortality rate (13.5% to 29%) (11,12). However, more recent Chinese reports on hospitalized cancer patients have shown that the average mortality rate of COVID-19 among cancer patients was closer to 5%–10% (13,14) and that patients with lung cancer or lung metastases were more prone to die from COVID-19 (13). A report from New York City suggested that only cancer patients younger than 50 years were at higher risk of death when in ICU (15). The UK National Health Service reported a very weak association between cancer and the risk of dying from COVID-19 (16). Altogether, these cohorts appeared to be heavily biased because they only included hospitalized patients and were therefore unlikely to reflect the real clinical outcome of COVID-19 in cancer patients.

The first proven case of COVID-19 in France was reported on January 24, 2020. The French hospital emergency response plan (Plan Blanc) and a nationwide lockdown were implemented on March 6, 2020. Institut Curie is a prominent comprehensive cancer center with 3 sites in Paris, Saint Cloud, and Orsay (Supplementary Figure 1, available online). In view of the urgent need for more data on COVID-19 in cancer patients, we set up an institutional registry at the beginning of the pandemic in France to more accurately determine the incidence of the disease and understand the outcomes in this fragile population, as well as identify specific risk factors for poor outcome. We prospectively registered all patients with confirmed or highly suspected COVID-19, including ambulatory patients with specific follow-up. From March 13 to May 1, 2020, 9842 cancer patients were referred at one of the Institut Curie facilities, of whom 7833 patients were receiving active therapy for any type of cancer, including breast cancer (50%), lung cancer (8%), gynecological cancer (8%), gastrointestinal cancer (7%), eye cancer (6%), head and neck cancer (6%), urological cancer (6%), hematological malignancy (5%), and sarcoma (5%). In this paper, we report the clinical characteristics and outcomes of SARS-CoV-2–infected patients treated or followed for cancer at Institut Curie as of June 15, 2020.

## Methods

### Registry Implementation and Data Collection

An institutional database was implemented to prospectively collect information concerning cancer patients treated or followed at Institut Curie with suspected or confirmed SARS-CoV-2 virus infection. This registry was approved by the Institut Curie institutional review board. No documentation or informed consent was required according to French regulations, because this study was strictly observational. Redcap software was used as data repository. Starting on March 13, 2020, we consecutively registered patients with symptoms suggestive of COVID-19 and/or confirmed COVID-19 by nasopharyngeal swab reverse transcription polymerase chain reaction (RT-PCR) and/or images suggestive of COVID-19 on chest computerized tomography (CT scan). Files of requests for RT-PCR were cross-checked to track possible missing patients. The design and reporting of this case-cohort study strictly complied with STROBE guidelines (17).

The main data recorded were as follows: cancer history (age, sex, primary tumor site, localized or advanced tumor); current cancer treatment intent according to 3 categories (curative intent for adjuvant or neoadjuvant or early stage treatment, disease control when active cancer treatment was administered for advanced cancer, and palliative phase); COVID-19 features (symptoms; nasopharyngeal swab RT-PCR result, when performed; suggestive chest CT scan images, when performed; laboratory parameters); COVID-19 severity (ambulatory patients, hospitalization, transfer to ICU, COVID-19– or cancer-related death); consequences on current cancer treatment (delayed, canceled, modified). Comorbidities and comedications were also collected. Patients who received noninvasive respiratory support in conventional hospitalization wards were considered to be hospitalized patients and not ICU patients. Treatments specifically administered for COVID-19 were also recorded. Systematic medical follow-up was performed at days 7, 14, and 28 after inclusion in the registry. Ethnic group analyses are not allowed per French regulations.

### COVID-19 Diagnosis

From March 13, 2020, a systematic screening comprising a questionnaire and systematic body temperature check was implemented at entrance of all our facilities. According to national guidelines, SARS-CoV-2 RNA testing on nasopharyngeal swab (RT-PCR testing) was initially restricted to health-care workers and severely ill patients (18). Tests became more readily available (upon prescription) after March 25, 2020, and patients with COVID-19 symptoms were then tested for SARS-CoV-2 RNA whenever possible. SARS-CoV-2 RNA testing also became mandatory for all patients, even asymptomatic, scheduled for surgery at Institut Curie. Cancer care, imaging, and laboratory assessments are detailed in the Supplementary Methods (available online).

### Eligibility

Registry inclusion criteria were patients either systematically screened for SARS-CoV-2 infection, or currently under treatment or surveillance at the Institut Curie for a solid tumor or hematological malignancy, presenting any symptom of COVID-19, or with confirmed COVID-19 by nasopharyngeal swab RT-PCR, or presenting chest CT scan images suggestive of COVID-19. Only patients with positive imaging and/or positive RT-PCR testing were considered positive for COVID-19 for the present analysis, regardless of their symptoms.

### Study Objectives

The primary objectives of the study were to evaluate the incidence of COVID-19 in Institut Curie patients and describe the clinical presentation, management, and outcome of COVID-19 during the 28 days following onset of the first symptoms. Secondary objectives were to evaluate the impact of COVID-19 on cancer care and factors associated with COVID-19 severity, such as concomitant treatments or comorbidities.

### Statistical Analysis

Descriptive statistics were reported in all COVID-19 patients and in the subgroup with both positive RT-PCR and chest CT scan. Laboratory measurements at diagnosis were compared with the reference value measured during the 2 precedent months with paired student *t* tests. The main clinical outcome was defined as death due to COVID-19 and/or ICU admission during follow-up within 6 weeks postdiagnosis. Because 28-days follow-up was obtained in all but 4 patients, the outcome was treated as binary. Univariate and multivariate prognostic factor analysis was performed using a logistic model and the Wald statistics for tests of parameters. For the multivariate analysis, a backward selection model was applied starting from all variables statistically significant at the 10% level in the univariate analysis and with less than 10% of missing data. Sensitivity analyses were performed on the prognostic factors for death alone and for time to worsening (ICU or death) using log-rank test and semiparametric model after checking the proportional hazard assumption. All analyses were 2-sided and performed with SAS v9.4 software. A *P* value of less than .05 was considered statistically significant.

## Results

### Population and Symptoms

**Figure 1.**
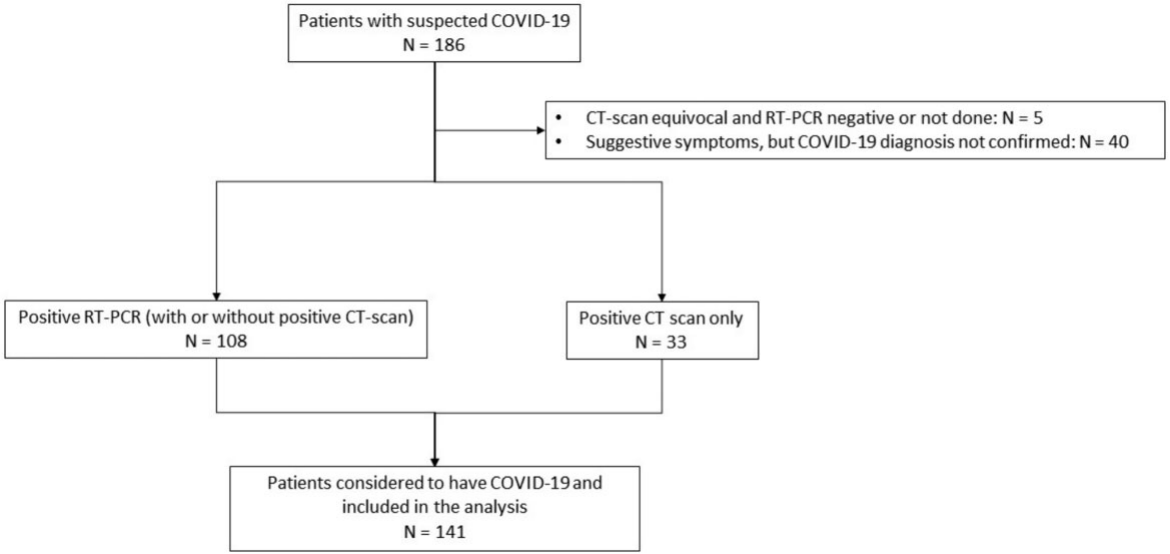
Flow chart. Figure showing how patients were selected for the present analysis. CT = computed tomography; RT-PCR = reverse transcriptase polymerase chain reaction.

From March 13, 2020, to April 25, 2020, 186 patients were entered into the registry. Forty-five patients were subsequently excluded because of uncertain diagnosis, leaving 141 patients with positive RT-PCR and/or positive chest CT scan for the final analysis (Figure 1). The weekly number of tested patients is illustrated in Supplementary Figure 2 (available online). Considering the 9842 patients who attended the Institut Curie at least once during the same period, the minimum estimated incidence of COVID-19 in our patients was 1.4%. Patient characteristics are detailed in Table 1, pointing to some numerical differences according to initial presentation. Overall, median age was 62 years, and 37 (26.2%) patients were older than 70 years. Of note, 25 (17.9%) patients were obese. Main preexisting comorbidities deemed relevant in the context of COVID-19 were hypertension (34.0%), smoking (17.7%), and diabetes (17.0%). The most common comedications were anticoagulants (24.1%), angiotensin-converting enzyme inhibitors and angiotensin II receptor blockers (20.6%), and corticosteroids (18.4%).

**Table 1.** Population characteristics

Characteristics	All patients No. evaluable (%) (n = 141)	RT-PCR^a^ No. evaluable (%) (n = 108)
Patients characteristics		
Median age, y (IQR)	62 (52-72)	63 (51-75)
Age >70 y	37 (26.2)	33 (30.6)
Gender, No.	141	108
Male	39 (27.6)	29 (26.8)
Female	102 (72.3)	79 (73.1)
BMI, No.	139	106
Median BMI,(IQR), kg/m^2^	25 (22-29)	25 (22-29)
BMI >30 kg/m^2^	25 (17.9)	18 (16.9)
WHO performance status, No.	83	64
0-1	42 (50.6)	28 (43.7)
2 or more	41 (49.4)	36 (56.3)
Comorbidities, No.	141	108
Active smokers	25 (17.7)	21 (19.4)
Chronic lung disease	7 (4.9)	5 (4.6)
Diabetes	24 (17.0)	20 (18.5)
Hypertension	48 (34.0)	43 (39.8)
Other heart disease	21 (14.9)	18 (16.7)
Systemic disease	6 (4.2)	5 (4.6)
None of the above	88 (62.4)	72 (66.7)
Concomitant medications, No.	141	108
Corticosteroids^b^	26 (18.4)	18 (16.7)
NSAID	8 (6.4)	7 (6.5)
ACE inhibitor/ARB	29 (20.6)	27 (25.0)
Anticoagulants	34 (24.1)	27 (25.0)
Immunosuppressants	6 (4.2)	4 (3.7)
Inpatients at diagnosis, No.	141	108
Yes	34 (24.1)	32 (29.6)
No	107 (76.9)	76 (70.4)
Cancer characteristics		
Site of cancer, No.	141	108
Breast	57 (40.4)	42 (38.9)
Hematological	19 (13.5)	16 (14.8)
Lung	18 (12.8)	11 (10.2)
Gynecological	12 (8.5)	10 (9.3)
Gastrointestinal	11 (7.8)	8 (7.4)
Head and neck	8 (5.7)	8 (7.4)
Sarcoma	6 (4.2)	5 (4.6)
Uveal melanoma	4 (2.8)	3 (2.8)
Genitourinary	2 (1.4)	2 (1.8)
Brain tumors, CNS	2 (1.4)	2 (1.8)
Other	2 (1.4)	1 (0.9)
Disease stage (solid tumors), No.	122	92
Localized	38 (31.1)	35 (38.1)
Advanced/metastatic	84 (68.9)	57 (61.9)
Pleuropulmonary metastases (solid tumors)	95 39 (41%)	66 22 (33%)
No. Metastatic sites, No.	119	89
0	35 (29.4)	32 (35.9)
1-2	71 (59.7)	52 (58.4)
≥3	13 (10.9)	5 (5.6)
Lines of treatments, No.	122	92
Adjuvant/neoadjuvant	38 (31.1)	35 (38.0)
Metastatic : 1-2 lines	20 (16.4)	14 (15.2)
Metastatic: ≥3 lines	64 (52.5)	44 (47.8)
Therapeutic intent on inclusion, No.	140	107
Curative	55 (39.3)	46 (42.9)
Disease control	51 (41.8)	37 (34.6)
Palliative	34 (27.9)	24 (22.4)
Ongoing cancer therapy,^c^ No.	141	108
Surgery	11 (7.8)	10 (9.3)
Chemotherapy	69 (48.9)	50 (46.3)
Radiation therapy	13 (9.2)	11 (10.2)
Endocrine therapy	21 (14.9	15 (13.9)
Targeted therapy	22 (15.6)	15 (13.9)
Immunotherapy	8 (5.7)	7 (6.5)
None	17 (12.1)	15 (13.9)
COVID-19–related symptoms and signs on inclusion in the registry		
Fever, ≥38.0°C	75 (53.2)	63 (58.3)
Cough	52 (36.9)	42 (38.9)
Dyspnea	42 (29.8)	33 (30.6)
Decreased O_2_ saturation, <96%	16 (11.4)	12 (11.1)
GI disorders	12 (8.5)	10 (9.3)
Anosmia/dysgeusia	10 (7.1)	7 (6.5)
Headache	4 (2.8)	4 (3.7)
No symptom	30 (21.3)	15 (13.9)

a RT-PCR: refers to the subset of patients with positive SARS-Cov2 RNA RT-PCR testing, whatever the imaging data. ACE = angiotensin-converting enzyme; ARB = angiotensin II receptor blocker; BMI = body mass index; CNS = central nervous system; IQR = interquartile range; NSAID = nonsteroidal anti-inflammatory drugs; RT-PCR = reverse transcriptase polymerase chain reaction; WHO = World Health Organization.

b Corticosteroids: We excluded corticosteroids administered in combination with chemotherapy and only included long-term corticosteroid therapy, usually prescribed in the context of advanced cancer.

c Ongoing cancer therapy refers to the cancer treatments received in the past 30 days.

Breast cancer was the most common cancer type (40.4%), followed by hematological malignancies (13.5%), lung cancer (12.8%), gynecological cancers (8.5%), and other miscellaneous tumors in line with the Institut Curie case mix. At the time of inclusion in the registry, 84 patients (68.9% of solid cancers) had advanced or metastatic cancer, including 39 patients (41.1%) with pleuropulmonary metastases. Seventeen patients (12.1%) were under surveillance for their cancer, and 124 (87.9%) were receiving ongoing treatment. Thirty-four patients (24.1%) had already been hospitalized for cancer care for more than 2 days at the time of COVID-19 onset. Of the patients, 39.3% received treatment with curative intent (adjuvant or neoadjuvant or early stage), 41.8% received treatment for disease control, and 27.9% received palliative care. Of note, these figures are very close to the general population of patients treated at Institut Curie during the same period of time. Accordingly, women account for 72.3% of this general population, who presented with a younger median age (57 years).

COVID-19–related clinical symptoms on inclusion in the registry were observed in 111 (78.7%) of the 141 patients. Fever (≥38°C) and cough were the most common symptoms (53.2% and 36.9%, respectively). Dyspnea was observed in 29.8% of patients, including 10.2% with respiratory distress. Other less common symptoms are shown in Table 1. A total of 14 COVID-19 patients had been identified through the systematic screening before initiation of treatment. They had either no symptoms (n = 10) or nonspecific symptoms such as fever pain and fatigue (n = 3); 1 had pulmonary distress. Suspicious imaging was found in 2 cases. Chemotherapy was delayed or modified in 4 of the 5 patients with an indication of chemotherapy. Surgery was delayed in 7 of the 8 patients with planned intervention. One of the 14 patients, who had gastroesophagus disease and lung metastasis and had more than 3 lines of prior treatments, was hospitalized and died of complications possibly related to COVID-19. All other patients recovered without admission to ICU.

### Imaging and Laboratory Findings

**Figure 2.**
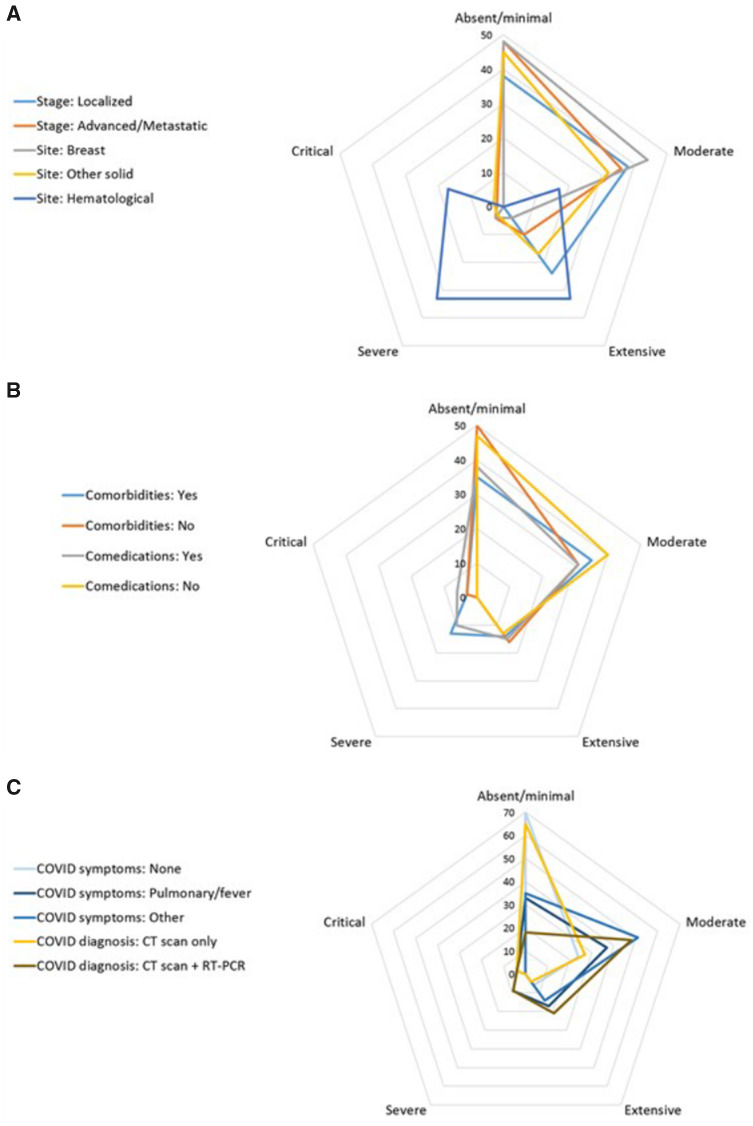
Baseline COVID-19–related findings on chest CT scan according to main baseline characteristics in 80 patients. Distribution of the initial extent of pulmonary parenchymal lesions is displayed according to cancer-related **(A)**, patient-related **(B)**, or COVID-19 diagnosis-related **(C)** parameters. Numbers indicate the percentage of each imaging pattern according to the 5-class classification we retained: absent/minimal (0% to <10%); moderate (10%-25%); extensive (26%-50%); severe (51%-75%); critical (>75%). The longest spokes indicate the highest frequency of the corresponding pattern. CT = computerized tomodensitometry.

Baseline chest CT scan findings, available for 80 patients, are described according to the extent of pulmonary parenchyma COVID-19–related alterations and clinical findings. The extent of parenchymal disease appeared to be relatively independent of the underlying patient or cancer key characteristics such as disease site or stage (Figure 2, A), comorbidities and comedications (Figure 2, B), or mode of COVID-19 diagnosis (Figure 2, C), although visual analysis suggested more extensive lung involvement in a small subset of patients with hematological malignancies. Detailed radiological findings are reported in Supplementary Table 1 (available online). Overall, extent of lung parenchyma involvement was no more than 50% in 95.8% of patients. Detailed radiological findings are reported in Supplementary Tables 1 and 2 (available online).

Laboratory test results (worse result at any time point) are detailed in Supplementary Table 2 (available online), and test results of special interest are shown in Table 2. We observed statistically significant, although clinically meaningless, differences in blood counts obtained in the same patients before and after SARS-Cov-2 infection. Statistically significant elevation of baseline serum C-reactive protein was recorded in 35 evaluable patients.

**Table 2.** Statistically significant laboratory findings at COVID-19 diagnosis compared with reference laboratory values

Biological values of interest	Reference^a^		At COVID-19 diagnosis		Mean of the differences		*P* ^b^
	No. evaluable	Median value (IQR)	No. evaluable	Median value (IQR)	No. evaluable	Mean value (IQR)	
Blood count							
Hemoglobin, g/dL	114	11.6 (10.5-13.2)	100	11.0 (9.2-12.4)	87	1.16 (-2.3 to +0.1)	<.001
Absolute neutrophil count, 10^9^/L	116	3.93 (2.59-5.81)	97	4.19 (2.52-7.32)	85	+0.77 (-1.72 to +1.95)	.42
Absolute lymphocyte count, 10^9^/L	114	1.18 (0.79-1.74)	96	0.83 (0.48-1.38)	82	−0.31 (-0.77 to +0.09)	.03
Platelets, 10^9^/L	114	240 (169-326)	100	190 (132-280)	87	−38.6 (-100 to +26)	.02
CRP, mg/L	46	20 (4.6-53.2)	71	84 (41-142)	35	+72 (+11 to +118)	.001

a Reference = laboratory test results obtained no more than 2 months before inclusion in the registry. CRP = C-reactive protein; IQR = interquartile range.

b Paired 2-sided student test.

### COVID-19 Therapy and Clinical Outcome

**Figure 3.**
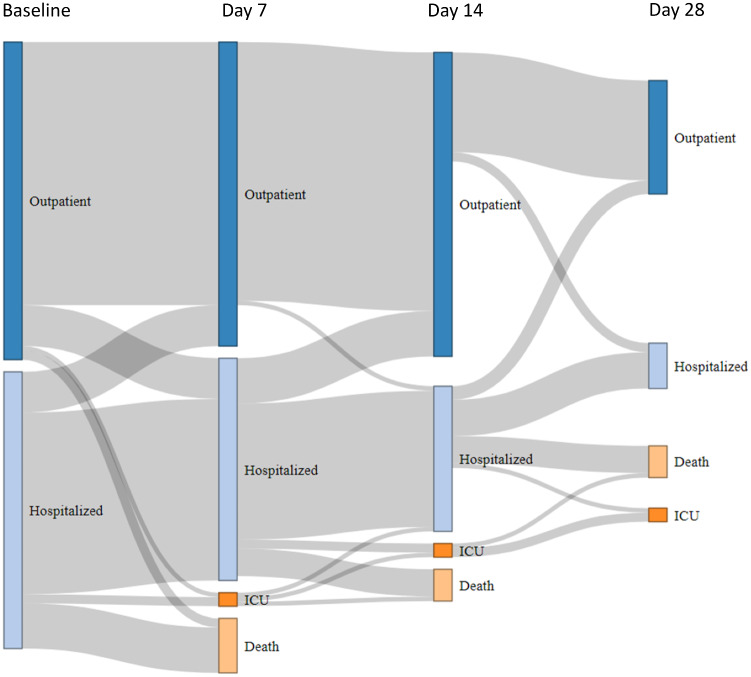
Patient trajectory from baseline to day 28. Diagram showing the changing status of cancer patients with COVID-19, based on individual follow-up. At baseline, patients were either immediately discharged home **(dark blue bars)** or hospitalized **(light blue bars)**, including patients already hospitalized at the time of COVID-19 diagnosis (hospital-acquired infection). The patient’s status may have changed on days 7, 14, and 28 to one of the following 4 modalities: discharged home **(dark blue bars)**, hospitalized **(light blue bars)**, admitted to the intensive care unit (ICU, **dark orange bars**), and deceased **(light orange bars)**. The **grey flows** between bars are proportional to the number of patients at each step.

Patients were either discharged home (64.2%) or hospitalized (35.8%) depending on the severity of the symptoms. Antibiotics were prescribed in 48.2% of patients according to local guidelines. Antiviral drugs were administered in selected cases (6.4%) after a thorough review of the patient’s medical history (Supplementary Table 3, available online). Most patients stayed at home and remained asymptomatic or very slightly symptomatic between baseline and day 28 (Figure 3). However, 11 (7.8%) patients were transferred to the ICU at some time during the course of the disease, and at the time of analysis, 26 patients (18.4%) had died from COVID-19, 4 (2.8%) had died from terminal cancer, 11 (7.8%) were recovering, and 100 (70.9%) were cured from COVID-19. Lung cancers had the worst outcome (n = 6, ie, 23.1% of all deaths) followed by hematological malignancies (n = 5, 19.2%), breast (n = 5, 19.2%), and gynecological cancers (n = 2, 7.7%). Twenty patients (76.9% of deceased patients) had received more than 3 lines of treatment for metastatic disease. The place of death was home (1 patient) or Institut Curie or other hospitals (12 and 8 patients, respectively) and was missing for 5 patients.

A total of 35 patients had poor outcome (transfer to ICU or death). On univariate analysis (Table 3), factors related to patient (aged older than 70 years, male gender), cancer (nonbreast cancer, palliative setting), or SARS-CoV-2 infection (pulmonary symptoms, extent of radiological signs of pneumonia) were statistically significantly associated with an increased risk of poor outcome. The final model of the multivariate analysis is provided in Table 3. A particularly interesting finding on multivariate analysis was that only a baseline decrease in O_2_ saturation and the extent of radiological signs of lung damage remained predictors of death and/or ICU admission, with odds ratios (ORs) of 6.71 (95% confidence interval [CI] = 1.40 to 30.10) and 2.48 (95% CI = 1.29 to 4.79), respectively. The odds ratios for age (older than 70 years) and nonbreast cancer were similar on univariate and multivariate analyses but were no longer statistically significant. Sensitivity analyses of the risk of death alone and for the time to death or ICU admission provided the same statistically significant associations.

**Table 3.** Analysis of prognostic factors associated with either ICU admission or death

Adverse prognostic factors	Univariate		Multivariate	
	OR (95% CI)	*P* ^a^	OR (95% CI)	*P* ^a^
Patient-related parameters				
Age, >70 y (vs ≤70)	2.44 (1.06 to 5.53)	.04	2.36 (0.55 to 9.65)	.21
WHO performance status, ≥2 (vs 0-1)	3.64 (1.18 to 10.12)	<.01	—	
BMI, ≥30 kg/m^2^ (vs <30)	0.57 (0.16 to 2.08)	.25	—	
Male (vs female)	2.57 (1.09 to 5.80)	.02	—	
Any comorbidity, Yes (vs No)	1.20 (0.48 to 2.66)	.64	—	
Hypertension, Yes (vs No)	1.88 (0.91 to 4.29)	.10	—	
Smoking, Yes (vs No)	0.85 (0.25 to 2.56)	.92	—	
Concomitant medications, Yes (vs No)				
Corticosteroids	1.45 (0.62 to 3.73)	.43		
NSAID	0.42 (0.09 to 3.53)	.42	—	
ACE inhibitor/ARB	1.49 (0.61 to 3.72)	.48	—	
Anticoagulants	1.99 (0.87 to 4.59)	.11	—	
Immunosuppressants	3.20 (0.60 to 17.10)	.16	——	
Inpatients infection (vs outpatient)	1.11 (0.54 to 2.75)	.80	—	
Cancer-related parameters				
Site				
Breast	1 (Referent)		1 (Referent)	
Nonbreast Lung Other solid tumor Hematological malignancy	3.64 (1.44 to 8.88) 3.55 (1.11 to 12.60) 4.51 (1.29 to 15.63) 3.26 (1.17 to 8.16)	<.01	3.55 (0.76 to 17.10) — — —	.12
Cancer stage (solid tumors)				
Advanced/metastatic (vs localized)	3.15 (1.00 to 10.25)	.05	—	
Pleuropulmonary metastasis (solid tumors)				
Yes (vs No)	1.04 (0.35 to 2.84)	.98	—	
Therapeutic intent				
Curative	1 (Referent)		—	
Control	1.75 (0.66 to 4.89)	.01^b^	—	
Palliative	4.65 (1.73 to 12.74)		—	
Line of treatment				
Neoadjuvant/Adjuvant (referent)	1 (Referent)		—	
Metastatic, ≤2 lines	2.11 (0.48 to 9.45)	.02^b^	—	
Metastatic, ≥3 lines	4.51 (1.42 to 14.31)		—	
COVID-19–related parameters				
Symptoms				
Dyspnea, Yes (vs No)	4.34 (1.93 to 9.63)	<.01	—	
Fever, Yes (vs No)	1.72 (0.81 to 3.73)	.19	—	
SpO_2_ <96%, Yes (vs No)	3.64 (1.34 to 10.67)	.02	6.71 (1.40 to 30.10)	.01
Radiology				
% alteration (per category)	2.39 (1.27 to 4.18)	.003	2.48 (1.29 to 4.79)	.004

a Wald statistics. All tests are 2-sided. ACE = angiotensin-converting enzyme; ARB = angiotensin II receptor blocker; BMI = body mass index; CI = confidence interval; ICU = intensive care unit; NSAID = nonsteroidal anti-inflammatory drugs; OR = odds ratio; SpO_2_ = oxygen saturation as detected by the pulse oximeter; WHO = World Health Organization.

b Global *P* values are presented.

### Impact on Cancer Care

Based on individual assessment, chemotherapy, targeted therapy, or immune checkpoint inhibitor therapy were delayed or stopped in 68.2%, 66.8%, and 83.1% of COVID-19 cases, respectively. Planned surgical procedures were postponed in 78.6% of cases. Ongoing radiation therapy was delayed or canceled in 69.4% of cases. Elective surgery was rescheduled after a mean of 3 weeks (15 days to 6 weeks).

## Discussion

The COVID-19 pandemic has and continues to have a dramatic impact on health-care systems worldwide. Cancer patients potentially represent one of the most vulnerable patient populations, and continuously updated data on how COVID-19 affects cancer care are essential. To the best of our knowledge, we report the first comprehensive prospective cohort of cancer patients, not only confined to hospitalized patients but also including outpatients with systematic and dedicated follow-up. We report 3 types of results.

Firstly, with 141 patients with COVID-19 observed during the period from March 13, 2020, to May 1, 2020, the minimum incidence rate of SARS-CoV-2 infection in the overall cohort of patients treated at Institut Curie during this period was 1.4% (or 1.7% when only considering patients with active cancer treatment). Although this incidence rate is potentially underestimated, it is not higher than the 5.7% rate estimated for the whole French population during the same period with the same diagnostic guidelines [ie, clinical screening, RT-PCR on nasopharyngeal swabs, and chest CT scan (19)]. Of note, strict distancing measures had been implemented in France by mid-March. We also implemented a distant warning system for all of our patients, including online messages, systematic text messaging, and e-mail warnings and recommendations. Furthermore, the proportion of infected people in the Paris area (Ile-de-France) in which the Institut Curie facilities are located (Supplementary Figure 1, available online) is estimated to be 12.3% (19), clearly suggesting that cancer patients, as a whole, are not more prone to SARS-CoV-2 infection than the general population when strict lockdown measures are implemented.

Secondly, the clinical features of COVID-19 in cancer patients appear to be very similar (Table 1 and Figure 2) to those observed in the general population (7,8,12). The vast majority of patients were symptom free and had been discharged home by day 28 (Figure 3), and no specific radiological (Figure 2;  Supplementary Table 3, available online) or laboratory (Table 2; Supplementary Table 2, available online) findings were observed. Furthermore, the mortality rate observed in our prospective cohort (19%) was also similar to that reported in the general population (up to 20%) (20), suggesting that the population of cancer patients presents a similar risk of dying from COVID-19 to that of the general population, in contrast with the results of several early retrospective studies (9,11,12,15). This is in line with the NHS report showing a slight increase of dying from COVID-19 limited to inpatients with recent diagnosis of cancer (16) and with the recent US report suggesting that risk of death is mostly due to general risk factors and some types of cancer such as hematological malignancies (21). On July 6, 2020, there were 29 920 COVID-19–related deaths reported and 168 335 confirmed COVID-19 diagnosis in France (5). The death rate was 17.8%, and this result is strikingly similar to the 18.4% death rate observed in our population. Interestingly, Kuderer et al. observed a death rate of 13% among their population of 928 patients with active or a history of cancer (21). Among them, 66% of patients had a performance status of 0 or 1 (compared with 50.6% in our population), 45% of patients presented remission or no evidence of disease, 32% has stable disease, and 22% had a progressive/metastatic disease (compared with 31.1% with localized or controlled disease and 68.9% of metastatic disease in our cohort). The general health state of patients in our cohort was more fragile, which may explain this difference. Another of the most striking observations in our cohort is that, when using ICU admission and/or death as a relevant clinical endpoint, COVID-19–related characteristics clearly appeared to be the most powerful predictors of poor outcome, as partly reported in several retrospective general cohorts (2,9,12). We observed a potential trend for a higher risk of death or ICU admission in older patients and in patients with nonbreast cancer (mostly lung and hematological malignancies) or in the palliative setting. Inpatients diagnosed with COVID-19 did not have a higher risk of death from the infection. Several hypotheses could also explain the lower-than-expected rate of severe COVID-19. In this study, in addition to hospitalized patients, we also included ambulatory patients, corresponding to patients with milder symptoms and a more favorable disease outcome. Of note, 24.1% of the patients of this cohort received anticoagulants, which may have prevented thromboembolic complications (22). We did not observe any case of cytokine storm syndrome (23), which may have been prevented by preexisting inflammatory state (24) or, conversely, immune exhaustion (25).

Thirdly, we also prospectively recorded the impact of COVID-19 on cancer care. More than two-thirds of scheduled cancer treatments were canceled, postponed, or modified. In a separate study, we evaluated that first consultations for breast cancer decreased by 34% compared with the same period in 2019 (26). As highlighted by many authors, the COVID-19 pandemic will very likely have a major collateral impact on cancer outcome in the coming months (27–31), while placing the cancer workforce under a considerable strain (32).

There are limitations to this study. Access to RT-PCR tests differed between the beginning and the end of the cohort registration period because of limited test availability at the beginning of the registration period (Supplementary Figure 2, available online). It is far too early to evaluate the actual cancer outcome of cancer patients in the COVID-19 era. We are also aware that breast cancer patients may be overrepresented in our cohort, which could contribute to the overall good prognosis of SARS-CoV-2 infection observed in this study. However, our practice is similar to that of other comprehensive cancer centers, and our experience can therefore provide useful information in this specific context. This registry needs to be continued to provide more extensive and robust data.

In summary, early reports on cancer and COVID-19, mostly based on hospitalized cancer patients, tended to suggest that cancer patients were more prone to suffer from SARS-CoV-2 infection and experienced a higher mortality rate. Special attention must be paid to patients with lung cancer and hematological malignancies. However, risk factors for COVID-19 mortality are mostly infection related rather than cancer related. Overall, our findings from a large prospective cohort of representative cancer patients and treatments, including ambulatory, nonhospitalized patients, strongly suggest that COVID-19 is neither more frequent nor more fatal in cancer patients as a whole. A simple baseline clinical assessment combining O_2_ saturation measure and a chest CT scan clearly identifies patients at risk of poor outcome and should be widely recommended in cancer patients with suspected COVID-19. As of April 28, 2020, systematic serological testing is now performed in all patients (those included in the present cohort as well as new patients). We believe that the data presented here, combined with more accurate epidemiological data based on longer follow-up and generalized antibody testing, will pave the way to safer and individualized care and lockdown ease for cancer patients in the COVID-19 era.

## Funding

This work was supported by Institut Curie, Université de Versailles Saint Quentin, and Université Paris-Saclay (no grant number applicable).

## Notes

**Role of the funder:** The funders had no role in the design of the study; the collection, analysis, and interpretation of the data; the writing of the manuscript; and the decision to submit the manuscript for publication.

**Disclosures:** The authors declare that they have no competing interests.

**Role of the authors:** CB, SD, VS, MF, AN, AB, PM, MAM, ASB, PV, SM, FCB, DV, TR, XP, LB, and PC contributed to data collection and interpretation. XP, LB, and PC set up the registry and contributed to data interpretation. CB, SD, MF, AN, and FCB contributed to manuscript writing. CB, SD, XP, LB, and PC collected data, contributed to the analysis, and wrote the manuscript. XP performed the statistical analyses. All authors read and approved the final manuscript.

**Acknowledgments:** Institut Curie COVID Group: Elisabeth Angellier, Muriel Belotti, Sara Boubakeur, Hervé Brisse, Bruno Buecher, Sylvie Carrié, Laetitia Chanas, Pascal Chérel, Gilles Créhange, Christelle Colas, Olivier Collin, Baudouin Courtier, Hélène Delhomelle, Emmanuelle Fourme, Thomas Frédéric-Moreau, Pierre Fumoleau, Marion Gauthier-Villars, Jean-David Heisbourg, Sophie Lassalle, Marine Le Mentec, Jane Muret, Sophie Métivier, Antoine de Pauw, Jean-Yves Pierga, Mael Priour, Roman Rouzier, Mary Saad, Claire Saule, Dominique Stoppa-Lyonnet, Anne Tardivon, Anne Vincent-Salomon.

## Data Availably

The data underlying this article cannot be shared publicly because of current French HIPAA regulations (birthdate, admission date, discharge date, date of death). Data will be shared on reasonable request to the corresponding author.

## Supplementary Material

pkaa090_Supplementary_Data
