# Supplementary material for: Characteristics and Outcome of SARS-CoV-2 Infection in Cancer Patients
Source: JNCI Cancer Spectr. 2021 Jan 6;5(1):pkaa090. doi: 10.1093/jncics/pkaa090 (PMC7665636; doi:10.1093/jncics/pkaa090)

SUPPLEMENTARY METHODS

Chest computed tomography-scan (CT-scan) protocol and image interpretation

Chest CT-scans were obtained with the patient in the supine position and with breath-holding following inspiration. Qualitative and semi-quantitative assessments were reached by consensus. In accordance with previous reports of COVID-19 imaging[1,2], the following features were recorded for each exam: ground-glass opacity (GGO), crazy paving, focal or linear consolidation. For each exam, the predominant semiological pattern was recorded. The presence of lung or pleural metastases was assessed by comparison with previous CT-scans. Patients with pre-existing equivocal findings before March 2020, such as GGO, were excluded after comparison of the study chest CT-scan with a previous CT-scan. A severity score (5 classes) was implemented according to the extent of lung parenchyma involvement[3]as follow : absent/minimal (0-<10%); moderate (10-25%); extensive (26-50%); severe (51-75%); critical (>75%).

Blood samples

Values of two samples tests were collected: one test called the reference test performed no more than two months before COVID-19 diagnosis and one test called COVID-19 diagnosis test performed between 7 days before COVID-19 diagnosis and 7 days after COVID-19 diagnosis. For the COVID-19 diagnosis test, most relevant values (meaning most abnormal values) were collected.

Cancer care during the COVID-19 pandemic

Starting mid-March 2020, national guidelines were published in France to help decision making for patients with cancer (fully accessible at https://www.oncopl.fr/recommandations-covid-19). These guidelines were similar for all tumor types and could be summarized as follows: surgery was to be postponed for localized stage when possible; medical cancer treatment could be delayed for at least 15 days or until symptoms resolution if COVID-19 disease was confirmed; and barrier gestures were strongly reminded and advised. Dedicated patients tracks were set up for radiation therapy in radiation therapy facilities for COVID-19 positive and negative patients. Hypofractionation

was preferred whenever possible[4]. Intensive care unit was organized with COVID positive and COVID negative areas and dedicated operating room (OR) was fitted with an airlock and air treatment. Surgery was maintained after tumor boards for curable solid tumor known to be resistant to chemotherapy and/or urgent surgical indication (e.g. sarcomas, melanomas, retinoblastomas, ovarian cancer, etc.) or breast cancer when surgery could not be delayed by neoadjuvant chemotherapy). In these cases, systematic COVID-19 screening was done and final decision was took by surgeons and anesthesiologists. Urgent surgery for COVID-19 positive patients were performed in a dedicated OR. All inpatients received anticoagulation therapy, either low weight molecular heparin initiated at entry, or as prescribed prior to hospitalization (refer to Table 1 in article).

References

1. Huang C, Wang Y, Li X, et al. Clinical features of patients infected with 2019 novel coronavirus in Wuhan, China. *The Lancet*. 2020;395(10223):497-506. doi:10.1016/S0140-6736(20)30183-5

2. Salehi S, Abedi A, Balakrishnan S, Gholamrezanezhad A. Coronavirus disease 2019 (COVID-19) imaging reporting and data system (COVID-RADS) and common lexicon: a proposal based on the imaging data of 37 studies. *Eur Radiol*. Published online April 28, 2020. doi:10.1007/s00330-020-06863-0

3. French Society of Radiology COVID-19 imaging guidelines. http://www.sfrnet.org/rc/org/sfrnet/nws/News/2020/20200316-155630-175/src/nws_fullText/fr/CR%20TYPE%20COVID-19%20LAST.pdf

4. Achard V, Tsoutsou P, Zilli T. Radiotherapy in the time of the Coronavirus pandemic: when less is better. *Int J Radiat Oncol Biol Phys*. Published online March 18, 2020. doi:10.1016/j.ijrobp.2020.03.008

SUPPLEMENTARY TABLES

Supplementary Table 1. Characteristics of patients with positive chest CT-scan.

**Characteristics of patients**	**No. evaluable (%)**
All patients with positive chest CT scan, No.	80
Median Age, y (IQR)	61 (52-77)
Age>70 y	18 (22.5%)
Gender	
Male	26 (32.5%)
Female	54 (67.5%)
Median BMI, kb/m^2 (IQR)	25 (22-29)
BMI≥30 kg/m^2	13 (16.3%)
WHO Performance Status (n=49)	
0-1	25 (51.1%)
2-4	24 (48.9%)
Comorbidities	
Active smokers	13 (16.3%)
Chronic lung disease	4 (5.0%)
Diabetes	14 (17.5%)
Hypertension	21 (26.3%)
Other heart disease	12 (15.0%)
Systemic disease	2 (2.5%)
None of the above	39 (48.7%)
Concomitant medications	
Corticosteroids	16 (20.0%)
NSAID	5 (6.3%)
ACE inhibitor / ARB	14 (17.5%)
Anticoagulants	22 (27.5%)
Immunosuppressants	4 (5.0%)
Inpatient infection	
Yes	17 (21.3%)
No	63 (78.7%)
Site of cancer	
Breast	32 (40.0%)
Hematological	10 (12.5%)
Lung	13 (16.3%)
Gynecological	6 (7.5%)
Gastrointestinal	9 (11.3%)
Head & Neck	4 (5.0%)
Sarcoma	3 (3.8%)
Uveal melanoma	2 (2.5%)
Genitourinary	0
Brain tumors, CNS	0
Other	1 (1.3%)

Disease stage (solid tumors, n=78)<br>Localized<br>Advanced/metastatic	 14 (17.9%)<br>64 (82.1%)
Pleuropulmonary metastases<br>(solid tumors, n=71)	 29 (40.8%)
Number of metastatic sites (n=79)<br>0<br>1-2<br>≥ 3	 21 (26.6%)<br>48 (60.7%)<br>10 (12.7%)
Number of line of treatments (n=78)<br>Adjuvant/neoadjuvant<br>Metastatic : 1-2 lines<br>Metastatic: ≥ 3 lines	 14 (17.9%)<br>13 (16.7%)<br>51 (65.4%)
Therapeutic intent at entry (n=80)<br>Curative<br>Disease control<br>Palliative	 24 (30.0%)<br>32 (40.0%)<br>24 (30.0%)
Ongoing cancer therapy (n=80)<br>Surgery<br>Chemotherapy<br>Radiation therapy<br>Endocrine therapy<br>Targeted therapy<br>Immunotherapy	 2 (2.5%)<br>45 (56.3%)<br>7 (8.8%)<br>14 (17.5%)<br>14 (17.5%)<br>6 (7.5%)
COVID-19 related symptoms and signs on inclusion in the registry	
Fever (≥38.0°C)	39 (48.8%)
Cough	28 (35.0%)
Dyspnea	27 (33.8%)
Decreased SpO_2 (<96%)	12 (15.0%)
GI disorders	8 (10.0%)
Anosmia/Dysgeusia	7 (8.8%)
Headache	0
No symptom	18 (22.5%)

Supplementary Table 2. Detailed CT scan pulmonary findings obtained from 71 patients[a]

Imaging semiology	Extent of lung damage on CT scan at diagnosis of COVID-19 N (%)				
	**Absent/ minimal (<10%)**	**Moderate (10-25%)**	**Extensive (26-50%)**	**Severe (51-75%)**	**Critical (>75%)**
Ground glass opacities	18 (81.8%)	13 (52.0%)	8 (80.0%)	2 (100%)	1 (100%)
Crazy paving	1 (4.6%)	6 (24.0%)	1 (10.0%)	0	0
Consolidation	3 (13.6%)	6 (24.0%)	1 (10.0%)	0	0

[a.] CT: computed tomography.

Supplementary Table 3. Detailed laboratory results. [a]

Laboratory values of interest	**No. evaluable**	**Median Values (IQR)**	**Abnormal, %**[b]
Hemoglobin, g/dL	127	10.7 (9.2 – 12.4)	72.2
Absolute Neutrophil count, 10^9/L	128	3.5 (2.3 – 5.0)	16.5
Absolute Lymphocyte count, 10^9/L	128	0.8 (0.5 – 1.3)	46.1
AST, IU/L	118	30.5 (24 – 50)	36.1
ALT, IU/L	118	29.5 (20 -45)	28.7
LDH, IU/L	59	311 (207 – 493)	36.6
PT, %	64	92.5 (78 – 100)	73.5
CRP, mg/L	82	66 (35 – 140)	94.2
PCT, mg/L	44	0.23 (0.1 – 0.7)	55.9

[a.] For each value of interest is shown the worst result, observed at any point during follow-up. AST: Aspartate transaminase. ALT: Alanine transaminase. LDH: Lactate dehydrogenase. PT: prothrombin time. CRP: C-reactive protein. PCT: procalcitonin.

[b] Abnormal values were defined as follows: for blood count and liver functional tests, according to NCI-CTC AE v4.0. For other biological values, we considered abnormal any value > 2 x Upper Normal Limit.

Supplementary Table 4. Antibiotics and anti-viral agents

**Type of treatment**	**No. (%)** N=141
Antibiotics	68 (48.2)
Corticosteroids	7 (4.9)
Hydroxychloroquine	5 (3.6)
Lopinavir/ritonavir	4 (2.8)

SUPPLEMENTARY FIGURES

Supplementary Figure 1. Paris Area “Ile-de-France” and location of Institut Curie facilities.

The seven Ile-de-France administrative boroughs (“départements”) are shown on the map, with their individual administrative numbers. The three locations of Institut Curie are shown in yellow (Core Paris), blue (Saint-Cloud, in the Hauts-de-Seine département) and red (Orsay, in the Essonne département).

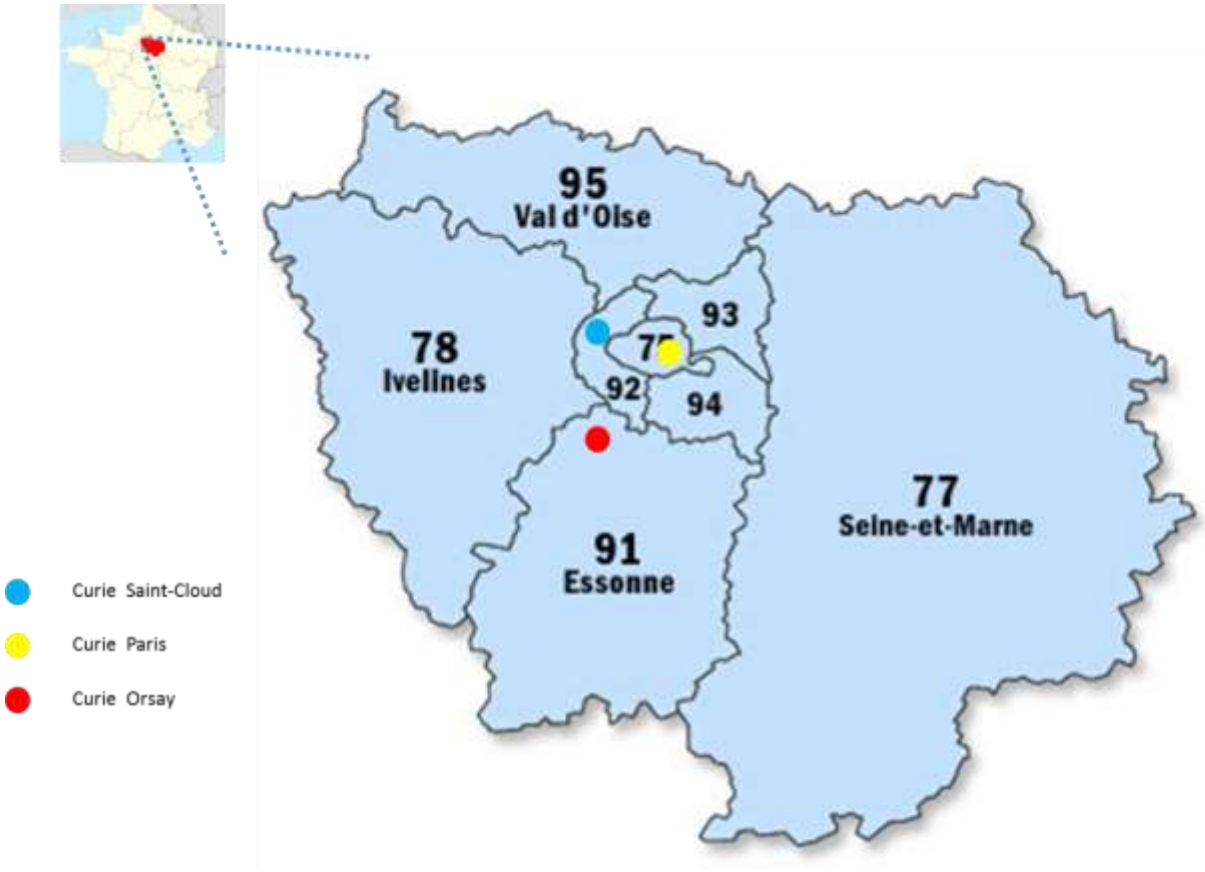

Supplementary Figure 2. Per week, number of tested, confirmed and hospitalized patients at Institut Curie.

The number per week of patients undergoing an RT-PCR test (blue curve), having a positive test (orange curve) or hospitalized (green curve) is indicated in the graph. Key events are indicated below the graph. Hospitalization was triggered only by the clinical condition and only symptomatic patients with positive RT-PCR testing or positive CT-scan imaging are represented in the figure.

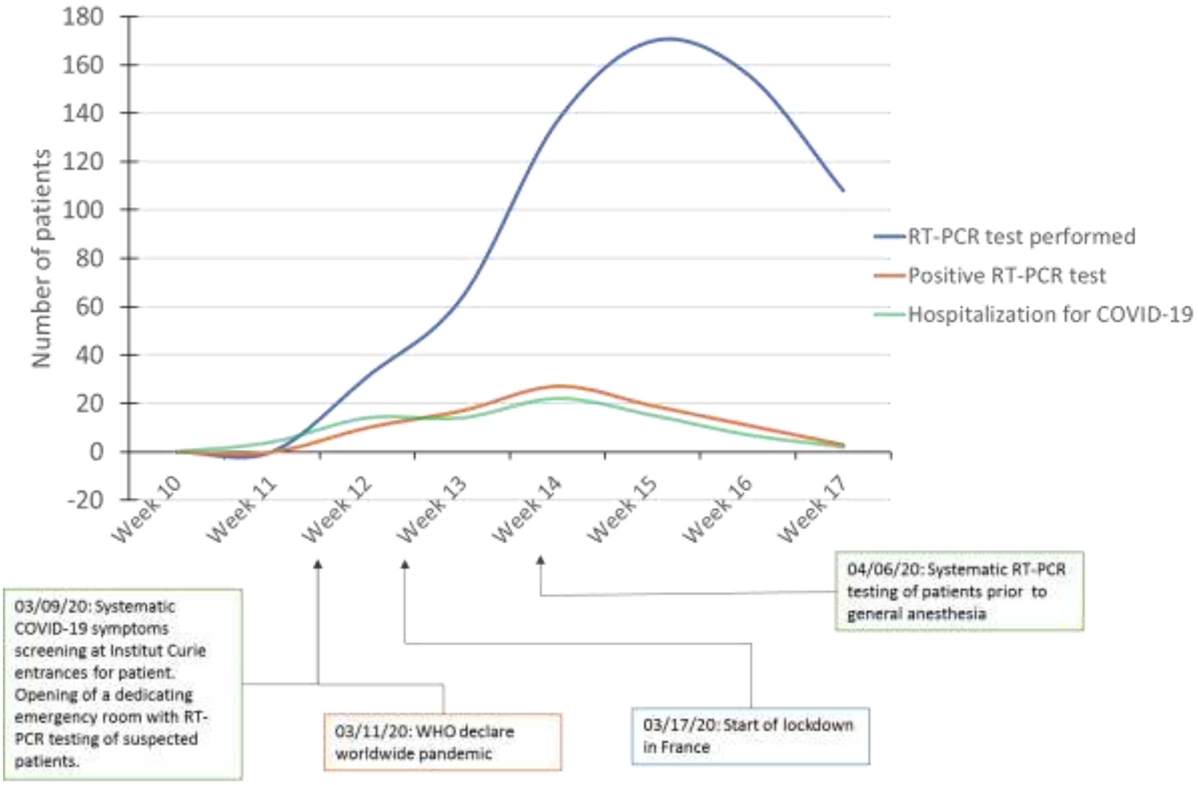

Supplement: pkaa090_Supplementary_Data [file pkaa090_supplementary_data.pdf]
